# Successful conservative management of a large 12-week-old cervical ectopic pregnancy in a nulliparous woman: a case report

**DOI:** 10.11604/pamj.2023.45.107.35701

**Published:** 2023-06-26

**Authors:** John Jude Kweku Annan, Stephen Ansah-Asamoah, Benedict Apaw Agyei, Eric Lartey Quarshie, Frank Ankobea, Sylvia Vanderpuye, Collins Oteng, Sebastian Yidana Ninimiya, Nana Esi Abedua Abaidoo

**Affiliations:** 1 Department of Obstetrics and Gynecology, School of Medicine and Dentistry, Komfo Anokye Teaching Hospital, Kwame Nkrumah University of Science and Technology, Kumasi, Ghana,; 2 Directorate of Obstetrics and Gynecology, Komfo Anokye Teaching Hospital, Kumasi, Ghana,; 3 Department of Medical Diagnostics (Medical Imaging Unit), Kwame Nkrumah University of Science and Technology, Kumasi, Ghana

**Keywords:** Cervical ectopic pregnancy (CEP), methotrexate, cervical encerclage tamponade, uterine-conserving, case report

## Abstract

Cervical ectopic pregnancy (CEP) accounts for less than 0.1% of all ectopic pregnancies. CEP is associated with high morbidity and mortality potential due to the associated life-threatening hemorrhage. When it is large, detected late, and occurs in a nulliparous woman, management is more challenging as it requires the need to preserve the uterus. We present a case of a 33-year-old nulliparous woman with a large live cervical ectopic pregnancy at 12 weeks + 1 day gestation and a very high serum β-HCG of 126,750 Miu/ml. She was successfully managed with suction curettage and cervical encerclage tamponade in order to preserve the uterus. The treatment was associated with significant hemorrhage and a prolonged period of follow-up. In low-resource settings, uterine-conserving management of CEP can be challenging, and curettage with cervical encerclage tamponade can be a cost-effective treatment modality even though it is associated with significant haemorrhage and prolonged treatment period.

## Introduction

Cervical ectopic pregnancy (CEP) is a rare form of ectopic pregnancy in which blastocyst implantation occurs in the endocervical canal rather than the uterine cavity [1]. They are traditionally considered extremely high risk as they may present with life-threatening unexpected bleeding due to erosion of the cervical blood vessels. As such, historically, they have been treated with hysterectomy, leading to loss of fertility. However, when detected early, fertility-preserving and uterine-conserving management approaches can be adopted. Medical approaches, such as the use of multidose systemic methotrexate (MTX) therapy with or without intra-amniotic potassium chloride (KCL) [2] and uterine-conserving surgical methods, such as suction curettage and cervical tamponade with either Foley´s catheter balloon, cervical cerclage sutures, and ligation of the cervical artery, have been used. Uterine artery embolization (UAE) [3] and hysteroscopic methods, although not readily available in low-resource settings, are novel uterine-preserving treatment approaches. Hysterectomy has traditionally been performed due to failed medical management or if the woman is hemodynamically unstable.

We report a case report of a large cervical ectopic pregnancy at 12 weeks gestation in a nulliparous woman. She had successful uterine-conserving management with suction curettage and cervical encerclage tamponade after failed medical treatment with methotrexate.

## Patient and observation

**Patient information:** a 33-year-old nulliparous woman, with a history of oligomenorrhoea, had a 3-month period of missed menses, during which she significantly experienced deep dyspareunia and pain on sitting down. There was no post-coital bleeding. An ultrasound scan to investigate the deep dyspareunia showed a suspected cervical ectopic pregnancy. She was referred to our tertiary facility for confirmation of the diagnosis and management. She had no significant medical history such as a history of sexually transmitted diseases, pelvic inflammatory disease, or uterine and cervical instrumentation. She had not used any form of contraception.

**Figure 1:**
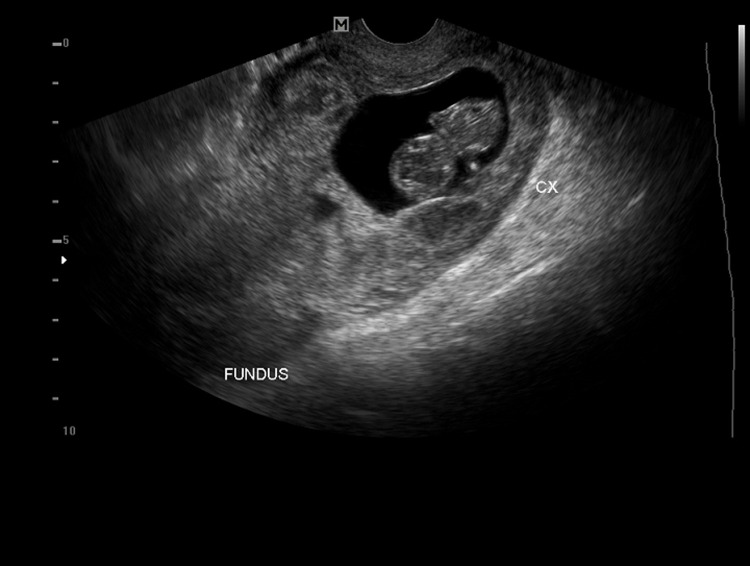
transvaginal ultrasound image of the uterus and cervix; the image shows an empty uterus; a gestational sac with a fetal pole is located in the endocervical canal

**Figure 2:**
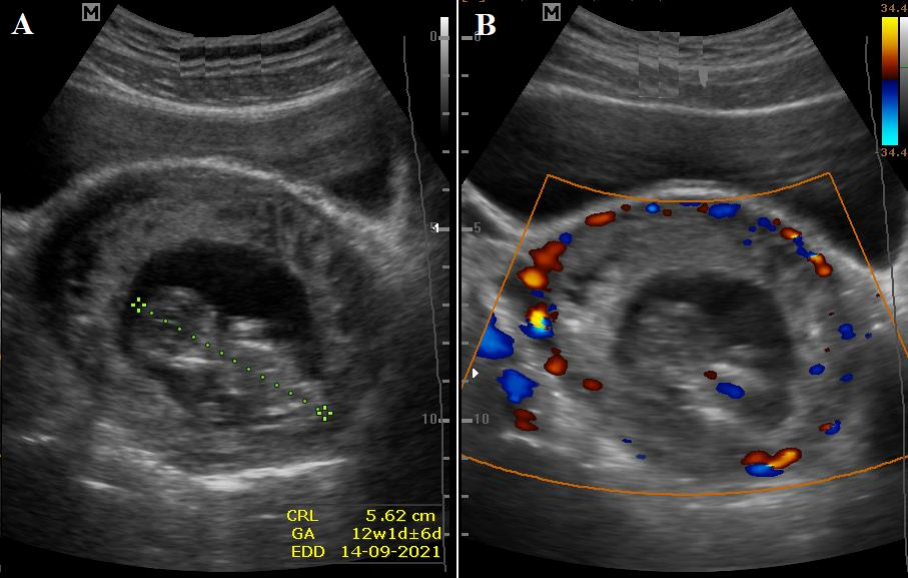
two transabdominal ultrasound images showing live cervical ectopic gestation: A) fetal pole with a Crown-rump length of 5.62 cm and a gestational age of 12 weeks+1 day; B) mass completely invaded both cervical lips and shows vascularity under Doppler color interrogation, fetal heart vascularity is seen

**Figure 3:**
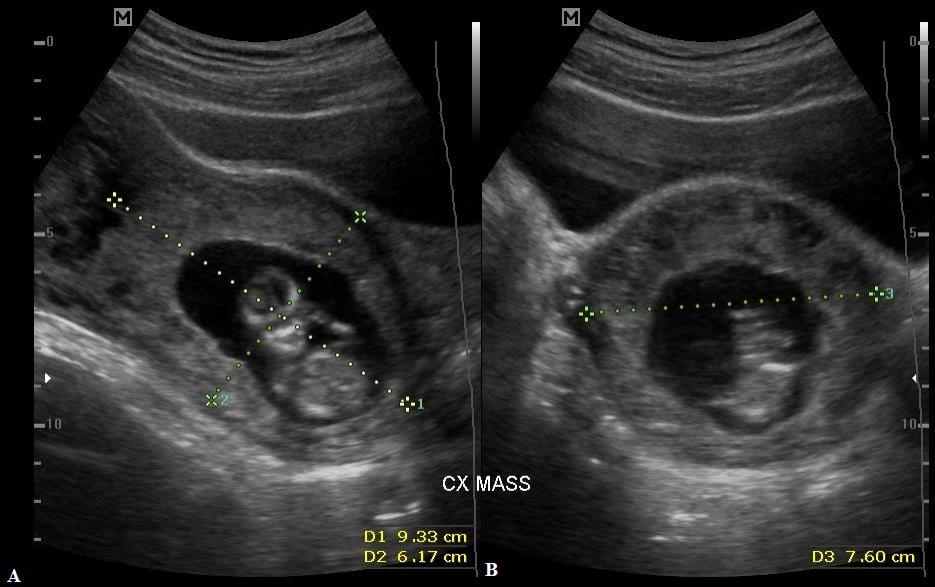
(A,B) transabdominal ultrasound image showing the cervical ectopic gestation measuring 9.33 cm x 7.60 cm x 6.17 cm; the pregnancy is restricted to the endocervical canal

**Clinical findings:** on clinical assessment. she was hemodynamically stable. Ultrasound scan showed a live CEP at 12 weeks + 1-day gestation, with the complete invasion of both cervical lips, and vascularity under Doppler color interrogation (Figure 1, Figure 2, Figure 3). Baseline laboratory results showed a hemoglobin concentration (Hb) of 12.6 g/dl, negative sickle cell screen, blood group of B Rhesus positive, and high serum β-HCG of 126,750 Miu/ml.

**Diagnostic assessment:** the couple was counselled on treatment options. She had read about CEP on the internet and requested a uterine-conserving approach since she was nulliparous and had fertility wishes. The unlikely success of medical management due to the nature of the CEP (large size, live ectopic, and high serum β-HCG) was explained to the couple. A decision for an attempt at the use of multi-dose methotrexate, with hysterectomy as the last option, in case of an intractable life-threatening haemorrhage was made. Pre-methotrexate evaluation with liver and renal function tests was performed, and the results were normal.

**Figure 4:**
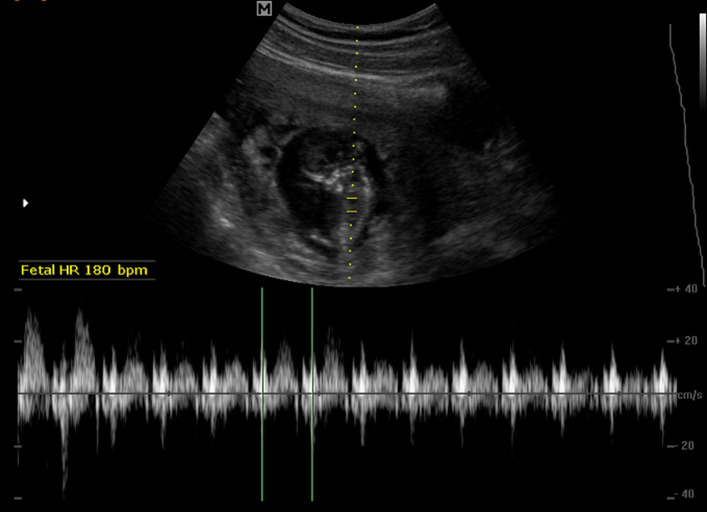
transabdominal scan image taken after completion of the multi-dose methotrexate treatment; the image shows persistent live cervical ectopic pregnancy with a fetal heart rate of 180 beats per minute

**Therapeutic intervention:** a week of multi-dose methotrexate injection was done. On day-7 post-methotrexate injection, serum β-HCG level was 125, 700 Miu/ml with a hemoglobin concentration of 12.0g/dl. Ultrasound scan showed a 10.9 x 7.5 x 6.1 cm CEP with positive cardiac activity but absent vascularity under Doppler interrogation (Figure 4). A decision was made for surgical intervention due to the size of the CEP and the static serum β-HCG level. Due to the unavailability of UAE facilities, suction curettage with cervical encerclage tamponade was recommended with hysterectomy as a lifesaving measure in case of a life-threatening hemorrhage. Under general anesthesia, two merselene tape cervical encerclage purse ring sutures were placed in situ, at an interval so that the sutures covered a greater portion of the area occupied by the CEP, but left loose and unknotted, with the aim of tying them immediately after the evacuation to arrest the expected torrential bleeding. Under transabdominal-ultrasound guidance, the evacuation was performed with suction curettage and the base of the crater was curetted. The two loose cervical encerclage sutures were tightly secured to arrest the bleeding. About 2.5 L of blood was lost during the suction curettage. The procedure took about 20 minutes. A check ultrasound scan showed a cervical hematoma of about 8 x 7 x 6 cm. Post-procedure, she was closely monitored with pad checks for bleeding and transabdominal scans for the size of the endocervical hematoma. Over a period of 3-hours, the hematoma size remained the same. Antibiotic prophylaxis, analgesia, and haematinics were administered. Post-evacuation Hb was 8.0g/dl. The tissue obtained from the evacuation was sent for histology. The serum β-HCG showed a significant reduction to 767.13 Miu/ml 48 hours post-evacuation. The cervical hematoma showed a reduction in size to 6.4 x 6.4 x 5.3cm (vol 114.2ml) on day 4. She was discharged home and weekly serum β-HCG assays were arranged with an advise to report immediately to the hospital in case she experienced significant vaginal bleeding.

**Figure 5:**
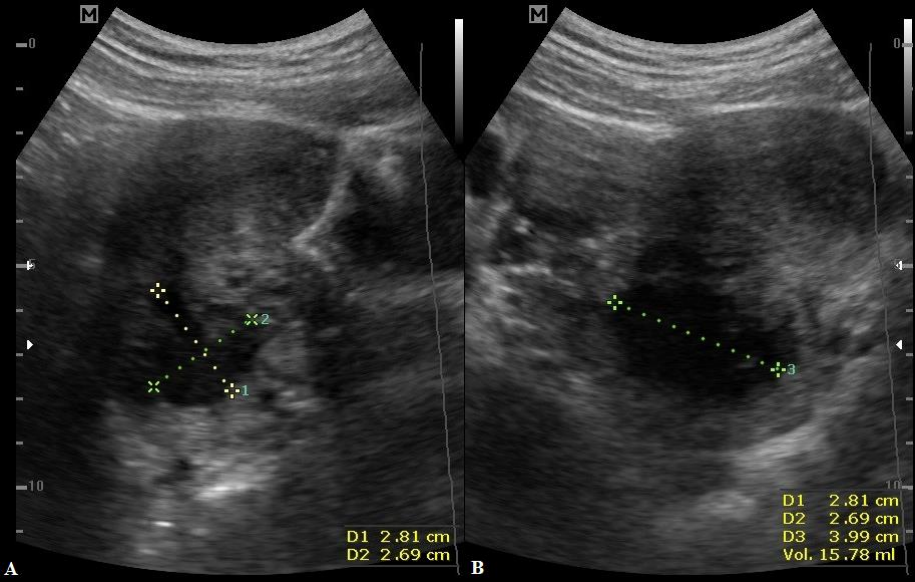
(A,B) transabdominal scan image taken 4 weeks after suction evacuation and cervical encerclage of the cervical ectopic pregnancy, showing absent fetal parts but a cervical collection measuring 2.8 x 2.6 x 3.9 (15.78ml)

**Follow-up and outcomes:** the histology report confirmed the presence of fetal parts with no evidence of malignancy. She had a prolonged period of follow-up. The serum β-HCG reduced to 104.56 Miu/ml on day-12, with a further significant reduction to 10.07 Miu/ml 4 weeks post-evacuation. The endocervical collection also showed a significant reduction from 6.0 x 6.0 x 5.1 cm (97.3 mls) to an insignificant size of 2.8 x 2.6 x 3.9 cm (15.78ml) over the period (Figure 5).

She remained afebrile but had mild spotting of a non-offensive brownish discharge. At 4 weeks + 2 days post-evacuation, her Hb was 12.8g/dl, and serum β-HCG was negative (3.85 Miu/ml). The cervical encerclage tamponade sutures were removed. The cervix looked normal in shape. There was no bleeding. Subsequently, she had her menses 5 weeks after the negative serum β-HCG result. She is currently planning another pregnancy.

**Patient perspective:** the patient expressed her indelible gratitude to the team for their professionalism and care. She was so pleased that her desire of preserving her womb was achieved so she could bear children. She expressed her desire to have the team take care of any subsequent pregnancy.

**Patient consent:** the patient provided written informed consent for the publication of this case report.

## Discussion

Cervical ectopic pregnancy (CEP) is the second-rarest form of ectopic pregnancy after abdominal pregnancy [1]. The predisposing factors include a history of pelvic inflammatory disease, endometritis, previous cervical surgeries, use of intrauterine devices, anatomic anomalies, and in vitro fertilization [1]. Interestingly, our patient did not have any of these risk factors.

Cervical ectopic pregnancy (CEP) has no specific clinical presentation. Our patient had unusual deep dyspareunia and pain on sitting down. But for the ultrasound scan, the diagnosis could have been delayed with a potential adverse outcome. Therefore, in sexually active women with oligomenorrhoea, a pregnancy test is recommended for the exclusion of pregnancy as part of the clinical workup. Ultrasonography established the definitive diagnosis. Her ultrasound features were consistent with the diagnostic criteria for CEP described by Jurkovic *et al*. (1996): (1) empty uterine cavity or thickened endometrium; 2) distended and/or enlarged cervix; 3) gestational sac or placental tissue below the level of the internal os; 4) negative sign of 'sliding organs'; and 5) high peri-trophoblastic vascularity on Doppler examination (peak velocity > 20 cm/s, pulsatility index < 1.0) [4].

Due to the associated life-threatening hemorrhage, large CEP has traditionally been treated with hysterectomy. However, with the advent of early pregnancy assessment units and improvements in ultrasound, early diagnosis is possible, allowing the use of conservative measures. A literature review showed case reports and case series that describe various conservative methods to successfully treat CEP. Treatment success is evidenced by a gradual fall in serum β-HCG until it becomes negative. These methods include transvaginal ultrasound-guided intra-amniotic and systemic methotrexate injection [2], high-intensity focused ultrasound (HIFU) followed by suction [5], transvaginal local injection of absolute ethanol [6], ultrasonographic-guided Laser Ablation [7], hysteroscopy [8], transcatheter intra-arterial methotrexate infusion combined with UAE followed by immediate curettage [3] and laparoscopic uterine artery clipping [9].

An initial failed attempt at medical management with parenteral multi-dose methotrexate was not surprising as the high serum β-HCG level, advanced gestation, and positive cardiac activity featured in reduced successful medical treatment. However, the patient´s request became a compelling factor to attempt this option. The uterine-conserving surgical method became inevitable, with hysterectomy as the last option in the event of intractable, life-threatening bleeding.

Curettage is a method for surgical excision of trophoblast tissue. Mechanical methods such as cervical tamponade using an inflated balloon or percutaneous embolization of pelvic vessels, and surgical ligation of cervical branches of uterine arteries are employed to minimize the associated hemorrhage.

A decision was made for curettage and cervical encerclage tamponade sutures since the other options were non-existent in our facility. To optimize the outcome, the following measures were instituted: availability of a considerable amount of blood products, the curettage procedure was done quickly, and preparation for prompt hysterectomy in the event of life-threatening hemorrhage. Significant hemorrhage signified by a reduction of Hb from 12.6g/dl to 8.0g/dl occurred even though the procedure was completed quickly in 20 minutes. Post-procedure, strict patient monitoring for vaginal bleeding and hemodynamic instability must be instituted. She remained stable. There was no significant increase in the size of the endocervical hematoma. When conservative management options are adopted, consideration must be given to the prolonged period for follow-up as it has significant financial implications (blood tests and scans) on the patient.

Uterine artery embolization (UAE) and curettage would have been the ideal treatment option due to the hypervascularity of this CEP. UAE has cost and affordability issues in low-resource settings, as well as a potentially adverse effect on fertility. The decision for primary hysterectomy must not be delayed in patients who present with intractable hemorrhage irrespective of fertility wishes. This helps to obviate emergency surgery and blood transfusion, especially in settings where access to blood products and transfusion services are difficult and in women who decline the use of blood products.

The lack of standardized care pathways for the management of CEP due to the rarity of CEP, presents a dilemma to the gynaecologist. The literature reports different successful CEP management intervention protocols. Albahlol IA, (2021) categorized a stepwise algorithm of successful interventions for CEP management to aid the practitioner [10]. Additionally, the Society of Obstetricians and Gynaecologists of Canada (SOGC) Clinical Practice Guideline No. 414 can serve as a useful adjunct when it comes to decision-making on the management of cervical ectopic pregnancies.

## Conclusion

We report a case of a large cervical ectopic pregnancy at 12 weeks gestation in a nulliparous woman who had successful uterine-conserving management with suction curettage and cervical encerclage tamponade. In low-resource settings, uterine-conserving management of CEP can be challenging, and curettage with cervical encerclage tamponade can be a cost-effective treatment modality even though it is associated with significant hemorrhage and prolonged treatment period.
